# miR-1224-5p Mediates Mitochondrial Damage to Affect Silica-Induced Pulmonary Fibrosis by Targeting BECN1

**DOI:** 10.3390/ijms18112357

**Published:** 2017-11-07

**Authors:** Qiuyun Wu, Tiantian Xu, Yi Liu, Yan Li, Jiali Yuan, Wenxi Yao, Qi Xu, Weiwen Yan, Chunhui Ni

**Affiliations:** 1 School of Public Health, Xuzhou Medical University, Xuzhou 221004, China; xjwqy922@163.com; 2 Department of Occupational Medicine and Environmental Health, Key Laboratory of Modern Toxicology of Ministry of Education, School of Public Health, Nanjing Medical University, Nanjing 211166, China; tiantianxutt@163.com (T.X.); liuyinjmu@163.com (Y.L.); liyan_njmu@163.com (Y.L.); yjlgogo@sohu.com (J.Y.); ywx3737@163.com (W.Y.); xuqi9876@126.com (Q.X.); weiwenyan911016@163.com (W.Y.)

**Keywords:** pulmonary fibrosis, silicosis, miR-1224-5p, BECN1, mitophagy

## Abstract

Silicosis is associated with fibroblast proliferation and extracellular matrix deposition in lung tissues. The dysregulation of miR-1224-5p has been implicated in several human cancers; however, the expression and function of miR-1224-5p in silicosis is unknown. The mitochondrial dysfunctions play critical roles in some diseases, but how these processes are regulated in silicosis remains limited. Here, we explored the role of miR-1224-5p in a mouse model of silicosis. We showed that the expression of miR-1224-5p is increased both in lung tissues of silica-induced pulmonary fibrosis and fibroblasts exposed to TGF-β1. Repression of miR-1224-5p expression attenuated silica-induced fibrotic progression in vivo and TGF-β1-induced myofibroblast differentiation in vitro. Additionally, we demonstrated that miR-1224-5p facilitated silica-induced pulmonary fibrosis primarily by repressing one of target genes, BECN1, thereby blocking PARK2 translocation to mitochondria and inducing the accumulation of damaged mitochondria. Furthermore, the activation of PDGFR signal mediated by mitochondrial damage and insufficient mitophagy resulted in myofibroblast differentiation. Collectively, these data indicated that miR-1224-5p exerts key functions in silica-induced pulmonary fibrosis and may represent a potential therapeutic target for silicosis.

## 1. Introduction

Silicosis, caused by prolonged exposure to silica dust, is one of the serious types of pneumoconiosis. Extensive fibroblast proliferation and deposition of extracellular matrix within the lungs are the typical characteristics [1,2]. Though the pathogenic factor is definite, it is not yet fully clarified the exact molecular mechanisms of the silicosis.

Healthy mitochondria play an important role in the maintenance of cell homeostasis. Mitochondrial dysfunction may have some unrecognized roles in the pathogenesis of silicosis. Mitophagy is the main type of autophagy and is known to degrade dysfunctional mitochondria [3]. Mitophagy appears to be involved in the development of several lung diseases, such as idiopathic pulmonary fibrosis (IPF) [4,5], chronic obstructive pulmonary disease (COPD) [6], and pulmonary hypertension (PH) [7]. However, few studies have been conducted to investigate the direct relationship among the mitochondrial dysfunction, mitophagy, and silicosis. Several studies indicated that silica disturbed mitochondrial energy generation and activated the mitochondrial apoptotic pathway in macrophage (RAW264.7) and epithelial cell (HBE, A549) [8,9,10]. Therefore, it is possible that aberrant mitochondria and mitophagy activity participate in the pathogenesis of silicosis, but the exact mechanisms by which mitophagy is modulated under silica exposure remains unknown.

MicroRNAs (miRNAs, miRs) get more attention as vital regulators for many cellular processes. Evidence suggests miRNA dysregulation is an important mechanism in pulmonary fibrosis. For instance, overexpression of miR-18a-5p inhibited bleomycin-induced pulmonary fibrosis in mice through reduction of TGF-βRII expression and suppression of TGF-β-Smad2/3 signaling [11]. So, further attentions are warranted to focus on the changes of miRNA expression in silica induced pulmonary fibrosis.

Our previous microarray analysis has suggested that miR-1224-5p is highly expressed in mouse lung tissues of silica-induced lung fibrosis [12]. miR-1224-5p has been demonstrated to be a negative regulator of TNF-α and is involved in the regulation of the LPS-mediated inflammatory responses [13]. Moreover, miR-1224-5p appears to target Parkinson’s disease-associated LRRK2 and α-synuclein genes [14]. In this study, we confirmed that miR-1224-5p expression was increased in mouse lung tissues of silica-induced pulmonary fibrosis compared with the mouse saline lung tissues. Repression of miR-1224-5p expression attenuated silica-induced pulmonary fibrosis both in vivo and in vitro by regulating its one of the target genes, BECN1, which is critical mediator in the PARK2 translocation to mitochondria and the degradation of damaged mitochondria. This study firstly reported that miR-1224-5p participates in silica-induced pulmonary fibrosis by directly repressing BECN1, thereby impairing mitochondria. Therefore, repression of miR-1224-5p expression might provide a novel therapeutic target of the silicosis.

## 2. Results

### 2.1. miR-1224-5p Is Increased and Mitophagy is Impaired in Mouse Lung Tissues in a Model of Silica-Induced Pulmonary Fibrosis

**Figure 1:**
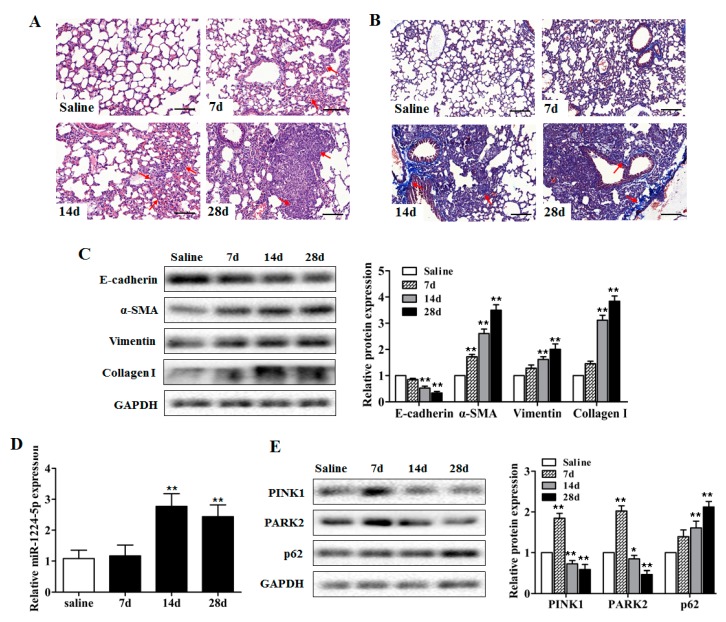
miR-1224-5p is increased and mitophagy is impaired in mouse lung tissues in a model of silica-induced pulmonary fibrosis. (**A**) The C57BL/6 mice were sacrificed on days 7, 14 and 28 after intratracheal instillation of silica suspended saline and saline. Histological changes of lung tissues were observed by haematoxylin and eosin (H&E) staining. Red arrows indicate the fibrotic nodules and damaged alveolar architecture. Scale bar: 100 µm; (**B**) Collagen deposition of lung tissues were observed by Masson’s trichrome staining. Red arrows indicate the collagen deposition. Scale bar: 100 µm; (**C**) Western blot and densitometric analysis of the protein expression of E-cadherin, α-SMA, Vimentin and Collagen I in mouse lung tissues, with ** *p* < 0.01 vs. the saline group; (**D**) qRT-PCR analysis of miR-1224-5p expression in mouse fibrotic lung tissue on days 7, 14 and 28 (*n* = 6 for each group), U6 was used as an internal control, with ** *p* < 0.01 vs. the saline group; (**E**) Western blot and densitometric analysis of the protein expression of PINK1, PARK2, and p62 in mouse lung tissues, with * *p* < 0.05 and ** *p* < 0.01 vs. the saline group. All data are expressed as the mean ± SD of at least three independent experiments.

Our previous miRNA microarray analysis of mouse lung tissues identified the significant up-regulation of miR-1224-5p in response to silica exposure (Supplementary Figure S1). However, the possible roles of miR-1224-5p in silicosis remain unclear. To determine if miR-1224-5p is specifically expressed in silicosis, we administered silica particles suspended in saline intratracheally to establish a mouse silicosis model. Histological changes via H&E staining and collagen deposition via Masson’s trichrome staining showed the destruction of alveolar architecture, collagen deposition, and local fibrotic nodules on day 28 after silica exposure (Figure 1A,B), which were further evidenced by decreased protein expression of epithelial cell marker (E-cadherin) and increased protein expression of mesenchymal cell markers (α-SMA, Vimentin, Collagen I) (Figure 1C, Supplementary Table S1). The expression levels of miR-1224-5p in mouse fibrotic lung tissues were verified by qRT-PCR method. Consistent with the microarray analysis, the miR-1224-5p levels on days 14 and 28 after silica exposure were significantly increased comparing with those in the saline group (Figure 1D).

Mitochondrial dysfunction has been shown to be an inducer that promotes PINK1 turning over at the mitochondrial outer membrane. This leads to the recruitment of the E3 ubiquitin ligase PARK2 to the mitochondria, which builds ubiquitin chains on mitochondrial proteins and activates mitophagy directly to remove damage mitochondria. The p62 is an important protein in mitophagy process, which will be degraded after the mitophagy finished completely [15]. Since the PINK1-PARK2 system and p62 have important roles in the selective degradation of damaged mitochondria by mitophagy, we intended to observe the protein levels of PINK1, PARK2, and p62 in fibrotic lung tissues. The PINK1 and PARK2 levels on day 7 after silica injection were increased then significantly decreased on days 14 and 28, and the p62 levels were significantly increased on days 14 and 28 compared with those in the saline group (Figure 1E and Supplementary Table S2). These data suggested that miR-1224-5p and mitochondrial damage may participate in silica-induced pulmonary fibrosis.

### 2.2. Down-Regulated miR-1224-5p Attenuates Silica-Induced Pulmonary Fibrosis and Restores Mitophagy In Vivo

**Figure 2:**
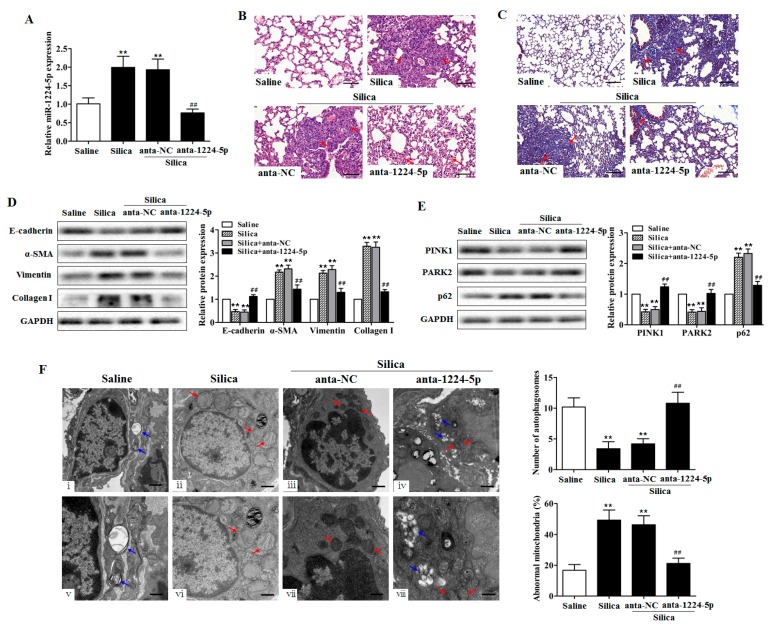
Down-regulated miR-1224-5p attenuates silica-induced pulmonary fibrosis and restores mitophagy in vivo. (**A**) qRT-PCR analysis of miR-1224-5p levels in mouse lung tissues after injection of saline, silica, silica plus antagomir negative control (anta-NC), and silica plus miR-1224-5p antagomir (anta-1224-5p) for 28 days, with ** *p* < 0.01 vs. the saline group and ^##^ *p* < 0.01 vs. the silica plus anta-NC group; (**B**) The lung histological lesions were observed with haematoxylin and eosin (H&E) staining. Red arrows indicate fibrotic foci and destruction of alveolar architecture. Scale bar: 100 µm; (**C**) Collagen deposition in lung tissues were observed by Masson’s trichrome staining. Red arrows indicate collagen deposition. Scale bar: 100 µm; (**D**) Western blot and densitometric analysis of E-cadherin, α-SMA, Vimentin and Collagen I expression in mouse lung tissues, with ** *p* < 0.01 vs. the saline group and ^##^ *p* < 0.01 vs. the silica plus anta-NC group; (**E**) Western blot and densitometric analysis of PINK1, PARK2, and p62 expression in mouse lung tissues, with ** *p* < 0.01 vs. the saline group and ^##^ *p* < 0.01 vs. the silica plus anta-NC group; (**F**) Transmission electron microscopy detection of mitochondrial structure and vacuoles of fibroblasts in mouse fibrotic lung tissues. Blue arrows indicate autophagic vacuoles. Red arrows indicate abnormal mitochondria. Quantification of autophagosomes and percentage of abnormal mitochondria (swollen with an irregular shape and disorganized cristae) from electron microscopy images were performed (five cells each group), with ** *p* < 0.01 vs. the saline group and ^##^ *p* < 0.01 vs. the silica plus anta-NC group. Scale bars, 1 µm (upper panels) and 500 nm (lower panels). All data are expressed as the mean ± SD of at least three independent experiments.

To understand whether miR-1224-5p have potential function in the development of silicosis in vivo, we conditionally downregulated miR-1224-5p in lung tissues in silica-induced pulmonary fibrosis mouse models using antagomir (anta-1224-5p). miR-1224-5p antagomir or its negative control (anta-NC) was co-injected via intratracheal instillation for the first time and via the tail vein at 7, 14 and 21 days after silica treatment. Then, lung tissues were harvested at day 28. As expected, at day 28 after the silica injection, miR-1224-5p levels were significantly downregulated in mouse fibrotic lung tissues (Figure 2A). Histological examination showed attenuated pulmonary fibrosis after silica treatment in miR-1224-5p down-regulated group, as evidenced by reduced destruction of alveolar architecture, less severe fibrotic foci and decreased collagen deposition (Figure 2B,C). Concomitantly, we observed obvious amelioration both in the severity and distribution of lung lesions in the down-regulation of miR-1224-5p (*p* < 0.01) (Table 1). Furthermore, the reduced expression of miR-1224-5p resulted in a decrease of the protein expression of α-SMA, Vimentin and Collagen I and a recovery of the protein expression of E-cadherin (Figure 2D and Supplementary Table S3). Together, these observations suggested that the down-regulated miR-1224-5p could attenuate silica-induced pulmonary fibrosis in vivo.

To assess the impact of down-regulated miR-1224-5p on mitochondrial damage and mitophagy in vivo, we detected the protein expression of PINK1, PARK2, and p62 in lung tissues. miR-1224-5p down-regulated mice exhibited the restored PINK1 and PARK2 expression and an attenuation of p62 levels upon silica treatment (Figure 2E, Supplementary Table S4). To determine the effect of miR-1224-5p on mitochondrial damage and mitophagy visually, we used transmission electron microscopy (TEM) to examine the mitochondrial structure and autophagic vacuoles of fibroblasts in mouse fibrotic lung tissues. Silica treatment decreased the presence of vacuoles in lung fibroblasts compared with those in the saline controls (Figure 2F(ii,vi)). Conversely, fibroblasts from miR-1224-5p down-regulated mouse lung tissues had more visualized vacuoles (Figure 2F(iv,viii)). Furthermore, lung fibroblasts in silica-treated mice had accumulated enlarged mitochondria with irregular shape and disorganized cristae (Figure 2F(ii,vi)), whereas lung fibroblasts in miR-1224-5p down-regulated mice had nearly normal mitochondria (Figure 2F(iv,viii)). Together, these results indicated that down-regulated miR-1224-5p restores mitophagy, thus removing damaged mitochondria in vivo.

### 2.3. miR-1224-5p Suppresses Mitophagy by Targeting BECN1

**Figure 3:**
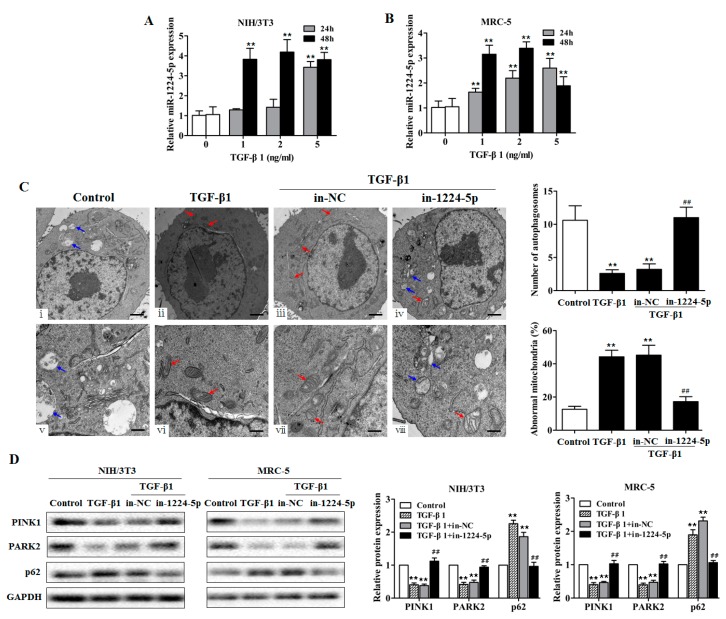
miR-1224-5p suppresses mitophagy in TGF-β1-exposed fibroblasts. (**A**,**B**) qRT-PCR analysis of miR-1224-5p levels in fibroblasts (NIH/3T3 and MRC-5) treated with different dose of TGF-β1 for 24 and 48 h, with ** *p* < 0.01 vs. the dose 0 group; (**C**) Transmission electron microscopy detection of mitochondrial structure and vacuoles of fibroblasts (NIH/3T3) transfected with miR-1224-5p inhibitor (in-1224-5p) or its negative control (in-NC) then treated with 2 ng/mL TGF-β1 for 48 h. Blue arrows indicate autophagic vacuoles. Red arrows indicate abnormal mitochondria. Quantification of autophagosomes and percentage of abnormal mitochondria (swollen with an irregular shape and disorganized cristae) from electron microscopy images were performed (five cells each group), with ** *p* < 0.01 vs. the control group and ^##^ *p* < 0.01 vs. the TGF-β1 plus in-NC group. Scale bars, 1 µm (upper panels) and 500 nm (lower panels); (**D**) Western blot and densitometry analysis of the protein expression of PINK1, PARK2, and p62 in fibroblasts (NIH/3T3 and MRC-5) transfected with in-1224-5p or in-NC, with ** *p* < 0.01 vs. the control group and ^##^ *p* < 0.01 vs. the TGF-β1 plus in-NC group. All data are expressed as the mean ± SD of at least three independent experiments.

In silicosis, activated macrophages can derive many cytokines including TGF-β1. TGF-β1 is recognized as a focus fibrogenic factor in pulmonary fibrosis, which could promote fibroblast proliferation and collagen deposition. Numerous studies employed TGF-β1 as the pulmonary fibrosis inducer in cell experiments [16,17]. In the present study, to better understand the biological function of miR-1224-5p, we treated fibroblast cell line (NIH/3T3, MRC-5) with various doses of TGF-β1 for 24 and 48 h. We observed that miR-1224-5p levels were significantly increased (Figure 3A,B), and α-SMA and Vimentin protein levels were increased compared with those in the control group (Figure S2A,B). In contrast, further analysis after miR-1224-5p inhibitor (in-1224-5p) transfection revealed significant reduction in the levels of α-SMA and Vimentin in fibroblasts (Supplementary Figure S2C,D).

To further examine mitochondrial damage and mitophagy activity in vitro, TGF-β1-exposed fibroblasts were visualized by transmission electron microscopy (TEM). In control fibroblasts, the existent number of vacuoles was observed in the cytoplasm (Figure 3C(i,v)). The TGF-β1-exposed fibroblasts exhibited nearly few vacuoles but accumulated aberrant mitochondria in the cytoplasm (Figure 3C(ii,vi)). The fibroblasts transfected with miR-1224-5p inhibitor resulted in restored mitophagy levels, in which the number of abnormal mitochondria were decreased and displayed some vacuolization in the cytoplasm (Figure 3C(iv,viii)). Consistent with the finding that down-regulated miR-1224-5p has a restored mitophagy levels in TEM analysis, fibroblasts transfected with miR-1224-5p inhibitor appeared to be increased PINK1 and PARK2 protein expression and decreased p62 levels after expose of TGF-β1 (Figure 3D and Supplementary Table S5).

**Figure 4:**
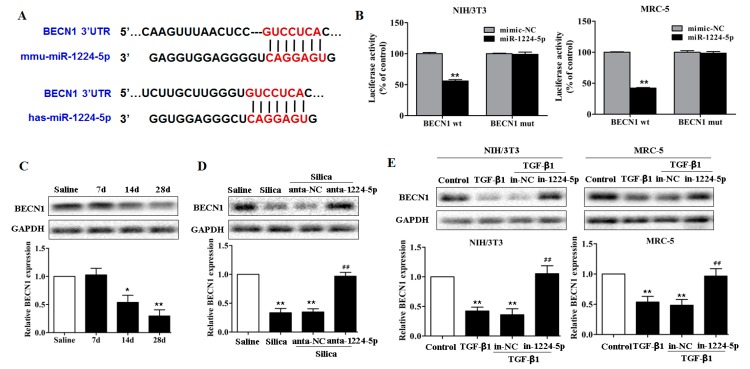
miR-1224-5p targets BECN1. (**A**) Schematic diagram of the conserved target sites of miR-1224-5p in the 3′UTR of human and mouse BECN1 mRNA. Seed sequences were indicated in red; (**B**) A luciferase reporter assays of the relative luciferase activity of fibroblasts (NIH/3T3 and MRC-5) transfected with BECN1-wt and BECN1-mut, with ** *p* < 0.01 vs. the miR-1224-5p mimic-NC group; (**C**) Western blot and densitometric analysis of BECN1 expression in mouse lung tissues on days 7, 14 and 28 after a single intratracheal instillation of saline or silica, with * *p* < 0.05 and ** *p* < 0.01 vs. the saline group; (**D**) Western blot and densitometric analysis of BECN1 expression in miR-1224-5p down-regulated mouse model, with ** *p* < 0.01 vs. the saline group and ^##^ *p* < 0.01 vs. the silica plus anta-NC group; (**E**) Western blot and densitometric analysis of BECN1 expression in fibroblasts (NIH/3T3 and MRC-5) transfected with in-1224-5p or in-NC, with ** *p* < 0.01 vs. the control group and ^##^ *p* < 0.01 vs. the TGF-β1 plus in-NC group. All data are expressed as the mean ± SD of at least three independent experiments.

The observation that damaged mitochondria and decreased mitophagy levels by miR-1224-5p promoted us to hypothesize that miR-1224-5p functions to suppress one or more key regulators of mitophagy, thereby maintaining the accumulation of abnormal mitochondria. To this end, we predicted the targets of miR-1224-5p according to their conserved binding sites. As shown in Figure 4A, miR-1224-5p was shown to bind to the 3′ untranslated region (UTR) of BECN1 mRNA, which is an important mitophagy molecule and play a central role in autophagosome formation and maturation. To test this, wild-type and mutant sequences of the 3′UTR of BECN1 were cloned downstream of a luciferase reporter, and these reporter constructs were co-transfected with miR-1224-5p mimic or mimic-NC into fibroblasts. In the presence of miR-1224-5p, the relative luciferase activity of wild-type BECN1 3′UTR was effectively reduced, whereas the BECN1-mut was unaffected (Figure 4B).

To explore whether BECN1 participates in silica-induced pulmonary fibrosis, we tested the BECN1 levels in fibrotic lung tissue and found that BECN1 exhibited a decreasing trend on day 14 and day 28 after silica treatment compared with those in the saline group (Figure 4C and Supplementary Table S6). In contrast, enhanced BECN1 protein expression was observed in the miR-1224-5p down-regulated mouse model (Figure 4D and Supplementary Table S7). To further explore the changes of BECN1 levels in fibroblasts in vitro, we performed Western blot analysis and observed that BECN1 were significantly decreased in fibroblasts treated with TGF-β1 (Figure 4E and Supplementary Table S8). Moreover, the transfection of miR-1224-5p inhibitor in fibroblasts induced restored BECN1 levels (Figure 4E and Supplementary Table S8). These data suggested that miR-1224-5p directly targets BECN1 and thereby inhibits the subsequent mitophagy signaling events.

### 2.4. BECN1 Facilitates PARK2 Translocation to Mitochondria

**Figure 5:**
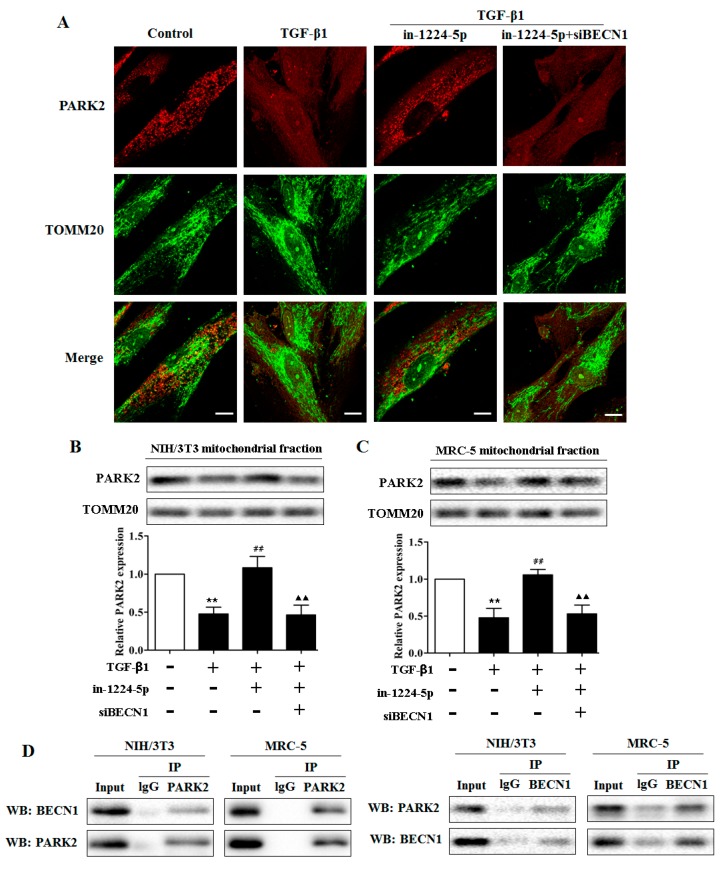
BECN1 facilitates PARK2 translocation to mitochondria. (**A**) Photographs of fluorescence staining by confocal microscopy evaluation in MRC-5 cells transfected with miR-1224-5p inhibitor (in-1224-5p) or co-transfected with in-1224-5p and BECN1 siRNA (siBECN1). TGF-β1 treatment (2 ng/mL) was started 24 h post-transfection and staining was performed after 48 h treatment. PARK2 expression was detected using an anti-PARK2 antibody and anti-TOMM20 antibody was used for mitochondria. Scale bar: 10 µm; (**B**,**C**) Western blot and densitometric analysis in in-1224-5p transfected or in-1224-5p and siBECN1 co-transfected fibroblasts (NIH/3T3 and MRC-5). TGF-β1 treatment (2 ng/mL) was started 24 h post-transfection and protein samples for mitochondrial fractions were collected after 48 h treatment, with ** *p* < 0.01 vs. the control group and ^##^ *p* < 0.01 vs. the TGF-β1 group and ^▲▲^ *p* < 0.01 vs. the TGF-β1 plus in-1224-5p group; (**D**) Endogenous protein-protein interactions between PARK2 and BECN1 in fibroblasts (NIH/3T3 and MRC-5) were determined by immunoprecipitation (IP) with PARK2 or BECN1 antibodies followed by Western blot analysis. IgG was used as negative control for IP. All data are expressed as the mean ± SD of at least three independent experiments.

Some research indicated that BECN1 promoted the PARK2 translocation to the mitochondrial fraction, which facilitates the mitophagy process and the remove of damage mitochondria [18,19]. To clarify if BECN1 has similar effects in the pathogenesis of silica-induced pulmonary fibrosis, and if miR-1224-5p interferes with these effects by targeting BECN1, fibroblasts were transfected with miR-1224-5p inhibitor alone or with BECN1 siRNA, then treated with 2 ng/mL TGF-β1 for 48 h. As shown in Figure 5A, confocal microscopy evaluation revealed a smaller proportion of PARK2 dots accumulation in TOMM20-stained mitochondria in TGF-β1-exposing cells compared with those in control cells. miR-1224-5p inhibitor transfected fibroblasts exhibited a significant increase in accumulation of PARK2 dots in TOMM20-stained mitochondria in response to TGF-β1 exposure. However, knockdown of BECN1 was able to reverse the effect of miR-1224-5p inhibitor-induced PARK2 dots accumulation. These may be related with the evidence that BECN1 is an important target of miR-1224-5p involved in the regulation of PARK2 translocation to the mitochondria.

To further test the hypothesis that miR-1224-5p suppresses PARK2 translocation then mitophagy by targeting BECN1 in silica-induced pulmonary fibrosis network, fibroblasts were also transfected with miR-1224-5p inhibitor alone or with BECN1 siRNA, and Western blot analyses were performed from mitochondrial fractions. It is showed that fibroblasts treated with TGF-β1 exhibited reduced PARK2 levels in mitochondria. In contrast, significantly increased PARK2 levels in mitochondria were observed in the miR-1224-5p inhibitor transfected cells, whereas the facilitative effect of miR-1224-5p inhibitor on PARK2 translocation was blocked by BECN1 siRNA transfection (Figure 5B,C and Supplementary Table S9). Thus, both microscopic and WB evidence suggested that miR-1224-5p knockdown promoted PARK2 translocation to mitochondria following TGF-β1 treatment whereas silencing target gene BECN1 inhibit PARK2 translocation in either case. Considering the role of BECN1 in PARK2 translocation, we next asked whether BECN1 interacts with PARK2. As shown in Figure 5D, the BECN1-PARK2 co-immunoprecipitation assays indicated the interaction between BECN1 and PARK2. Together, these experiments demonstrated that miR-1224-5p regulates mitophagy in a BECN1-dependent manner and that BECN1 regulates PARK2 translocation to mitochondria by interacting with PARK2. Thus, BECN1 appears to be involved in mitophagy and the remove of abnormal mitochondria thereby playing a crucial role in silica-induced pulmonary fibrosis.

### 2.5. PARK2 Knockdown Suppresses Mitophagy, Activates PDGFRs/PI3K/AKT Signaling Pathway and α-SMA Expression in Fibroblasts

**Figure 6:**
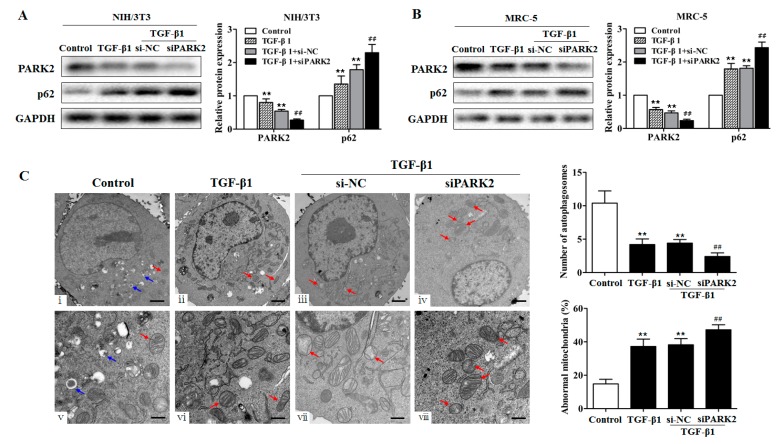
PARK2 knockdown suppresses mitophagy in fibroblasts. (**A**,**B**) Western blot and densitometric analysis of PARK2 and p62 expression in fibroblasts (NIH/3T3 and MRC-5) transfected with PARK2 siRNA (siPARK2) or its negative control (si-NC) then treated with 2 ng/mL TGF-β1 for 48 h, with ** *p* < 0.01 vs. the control group and ^##^ *p* < 0.01 vs. the TGF-β1 plus si-NC group; (**C**) Transmission electron microscopy detection of mitochondrial structure and vacuoles of fibroblasts (NIH/3T3) transfected with siPARK2 or si-NC then treated with 2 ng/mL TGF-β1 for 48 h. Blue arrows indicate autophagic vacuoles. Red arrows indicate abnormal mitochondria. Quantification of autophagosomes and percentage of abnormal mitochondria (swollen with an irregular shape and disorganized cristae) from electron microscopy images were performed (five cells each group), with ** *p* < 0.01 vs. the control group and ^##^ *p* < 0.01 vs. the TGF-β1 plus si-NC group. Scale bars, 1 µm (upper panels) and 500 nm (lower panels). All data are expressed as the mean ± SD of at least three independent experiments.

We next tested whether knockdown of PARK2 could result in the impairment of mitophagy in fibroblasts using a loss-of-function approach (employing PARK2 siRNA). In contrast to the control cells transfected with scramble siRNA (si-NC), cells transfected with PARK2 siRNA (siPARK2) exhibited the increased p62 expression (Figure 6A,B, Supplementary Table S10). To further examine mitochondrial damage and mitophagy activity, TGF-β1-exposed fibroblasts were visualized by transmission electron microscopy. The TGF-β1-exposed fibroblasts exhibited few vacuoles but accumulated enlarged swollen mitochondria in the cytoplasm compared with the control fibroblasts (Figure 6C(ii,vi)). Furthermore, both the accumulation of damaged mitochondria and the decrease of visualized vacuoles were more obvious after siPARK2 transfection (Figure 6C(iv,viii)). Together, these data indicated that knockdown of PARK2 was sufficient to induce fibroblasts to break normal mitophagy activity and accumulate damaged mitochondria.

**Figure 7:**
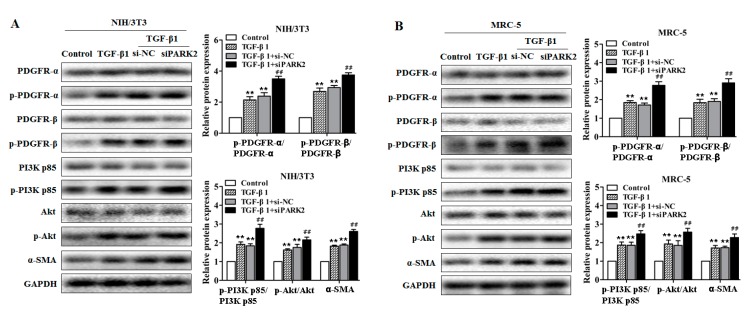

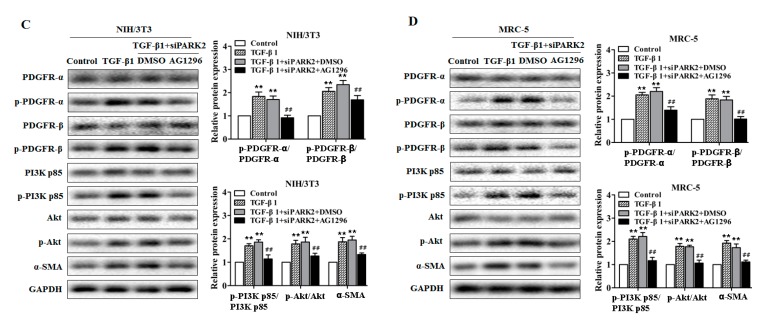
PARK2 knockdown activates PDGFRs/PI3K/AKT signaling pathway and α-SMA expression in fibroblasts. (**A**,**B**) Western blot analysis of PDGFRs/PI3K/AKT signaling pathway and α-SMA expression in fibroblasts (NIH/3T3 and MRC-5) transfected with siPARK2 or si-NC then treated with 2 ng/mL TGF-β1 for 48 h, with ** *p* < 0.01 vs. the control group and ^##^ *p* < 0.01 vs. the TGF-β1 plus si-NC group; (**C**,**D**) Western blot analysis of PDGFRs/PI3K/AKT signaling pathway and α-SMA expression in fibroblasts (NIH/3T3 and MRC-5) transfected with siPARK2, AG1296 (Abcam, ab141170, 15 mM) treatment was started at 12 h post-transfection then treated with 2 ng/mL TGF-β1 for 48 h, with ** *p* < 0.01 vs. the control group and ^##^ *p* < 0.01 vs. the TGF-β1 plus siPARK2 plus DMSO group. All data are expressed as the mean ± SD of at least three independent experiments.

Next, we examined the role of PARK2 knockdown in the activation of PDGFRs/PI3K/AKT signaling pathway and myofibroblast differentiation (α-SMA expression) in fibroblasts. As shown in Figure 7A,B, and Supplementary Tables S11 and S12), PARK2 knockdown in TGF-β1-treated fibroblasts led to the activation of PDGFRs/PI3K/AKT signaling pathway and the increase of α-SMA expression. To clarify whether PDGFR activation mediated by PARK2 knockdown is specifically responsible for α-SMA expression, we used AG1296, a PDGF receptor tyrosine kinase inhibitor, in PARK2 knockdown experiments. AG1296 efficiently inhibited phosphorylation of the PDGFRs/PI3K/AKT signaling pathway induced by PARK2 knockdown and TGF-β1 exposure, which was accompanied by decreased α-SMA expression (Figure 7C,D, and Supplementary Tables S13 and S14). These experiments demonstrated that the essential role of the PDGFRs signaling pathway activation mediated by insufficient mitophagy is mainly responsible for myofibroblast differentiation in fibroblasts.

## 3. Discussion

miRNAs are getting more attentions as critical regulators for pulmonary fibrosis processes, including silicosis. Here, we showed that the expression levels of miR-1224-5p are increased in fibrotic lung tissues in mouse models of silicosis. This suggested that miR-1224-5p-related dysfunction in the lung tissues may have an unrecognized role in the pathogenesis of silicosis. Our further studies indicated that the down-regulated miR-1224-5p could attenuate silica-induced pulmonary fibrosis in vivo. So the closer discussion of miR-1224-5p expression and function is needed to gain further insight into the roles of miR-1224-5p in the pathogenesis of silicosis.

Autophagy maintains cellular homeostatic balance by removing damaged proteins and organelles [20]. Some studies have indicated that autophagy can also contribute to the development of silicosis via regulating the function of macrophages and fibroblasts [21,22]. Mitochondria are known to regulate ATP generation for cell survival and functions, and its extracellular ATP and mitochondrial DNA release could cause inflammatory responses in some lung diseases, such as COPD and pulmonary fibrosis [23,24]. Mitophagy is implicated in selective elimination of damaged mitochondria by autophagosomes in order to maintain mitochondrial homeostasis [25]. Study showed that mitophagy, which is modulated by Akt1 activation, contributes to alveolar macrophage apoptosis resistance and is required for macrophage-derived TGF-β1 expression in the development of pulmonary fibrosis [26]. However, some evidence suggested that mitophagy is impaired in lung fibrosis. For example, mitophagy molecular PINK1 is decreased in type II alveolar epithelial cells from the IPF lung, and PINK1 reduction caused accumulation of damaged mitochondria, loss of cell viability, and activation of profibrotic responses [27]. There are significant discrepancies between these studies, likely due to different cell types as well as the heterogeneity of disease stages in pulmonary fibrosis. However, whether the mitochondria are damage in silicosis remains unknown. Our data showed that mitochondria are impaired in mouse lung tissues in a model of silica-induced pulmonary fibrosis and are restored in the miR-1224-5p down-regulated mouse model. It is possible that miR-1224-5p related regulation to silica-induced pulmonary fibrosis was associated with mitochondrial damage, which may be an important feature in the pathogenesis of silica-induced pulmonary fibrosis.

To date, only few miRNAs have been reported to modulate mitophagy. For example, miR-137 inhibits mitophagy via regulation of two mitophagy receptors FUNC1 and NIX [28]. miR-320a promotes mitophagy by down-regulating VDAC1 expression during serum starvation in cervical cancer cells [29]. Presently, the roles of miRNAs-regulated mitophagy in silicosis are poorly characterized. BECN1 is an autophagic protein, which involves in the class III PI3 kinase complex (PI3KC3) that induces the formation of autophagosomes. The decreased expression of BECN1 is detected in idiopathic pulmonary fibrosis fibroblasts [30]. In recent years, some studies elucidated that BECN1 also plays important roles in mitochondrial quality control by involving in the mitophagy process [31,32]. Here, we confirmed that miR-1224-5p exerts a specific regulation of BECN1. Inhibition of miR-1224-5p significantly increased the BECN1 protein levels under the silica stimulation. Therefore, our study may contribute to understanding the regulation mechanism of mitophagy by miRNAs in silica-induced pulmonary fibrosis.

PINK1-PARK2 system plays focus roles in the activation of mitophagy directly and the remove of damage mitochondria [33,34]. PARK2 dysfunction leads to progressive mitochondrial damage that may play a broader role in Parkinson’s disease [35]. Our data showed that PINK1 and PARK2 expression on day 7 after silica injection were increased then significantly decreased on days 14 and 28. This may explained that under silica treatment with an early stage, cells may induce mitophagy as a defense mechanism to remove damaged mitochondria accumulation. Under silica treatment for a long time, mitophagy defense mechanism may not be enough to degrade the damaged mitochondria and induced pulmonary fibrosis. Our molecular study further demonstrated that BECN1 interacts with PARK2, and BECN1 depletion inhibited PARK2 translocation to mitochondria in TGF-β1-treated fibroblasts. In line with our findings, several studies have indicated that BECN1 could influence the mitochondrial translocation of PARK2 [18,19]. Given the critical roles of mitochondria in cellular homeostasis, the insufficient mitophagy will cause continuous damage and aberrant accumulation of mitochondria [36]. In this study, we also observed that knockdown of PARK2 with specific siRNA results in apparent accumulation of damaged mitochondria with swelling and crista disruption in TGF-β1-treated fibroblasts and increased accumulation of p62 when autophagy flux was weakened. These data suggested that translational inhibition of BECN1 by miR-1224-5p blocks PARK2 translocation and thereby, inhibits cells from the maintaining of mitophagy activity under the silica stimulation.

Platelet-derived growth factors (PDGFs) and its receptors (PDGFRs) family can promote the proliferation of fibroblasts and deposition of extracellular matrix in cardiac fibrosis [37]. PDGF receptor inhibitors could attenuate radiation-induced pulmonary inflammation and fibrosis in C57BL/6 mice, which are also effective in inhibition of hepatic stellate cell proliferation and hepatic fibrogenesis [38,39]. In terms of clinical relevance of PDGFR signaling in IPF pathogenesis, one of the representative target receptors of nintedanib is PDGFR. In lung fibrosis animal models, PDGFR and its downstream PI3K/Akt play important roles in the proliferation, transformation, and collagen synthesis of fiber cells [40,41]. In this study, we explored whether the activation of PDGFR/PI3K/Akt signaling pathways is mediated by insufficient mitophagy and the relation of PARK2 to this role in TGF-β1-treated fibroblasts. Results showed that PARK2 knockdown in fibroblasts led to the PDGFRs/PI3K/AKT signaling pathway activation as well as α-SMA expression. After addition of AG1296, the PDGFR/PI3K/Akt signaling pathway was efficiently inhibited, accompanied by reduction in α-SMA expression. Consistent with the previously described study [5], these data suggested that the activation of PDGFR signal mediated by insufficient mitophagy results in myofibroblast differentiation. Despite an increased differentiation potential of fibroblasts by PDGFR signal, there may be other mechanisms that are involved in silica-induced pulmonary fibrosis in insufficient mitophagy mice, and the specific mechanism is required to be further studied.

In this study, miR-1224-5p antagomir was instilled directly through the trachea at the first time point, but then via the tail vein for three subsequent time points in order to decrease injury and promote blood absorption. miRNA antagomir injection via the tail vein was an efficient method to repress miRNA expression, but it is not yet clarified whether entire miR-1224-5p are repressed in any organs/tissues, which will be explored in our future study.

**Figure 8:**
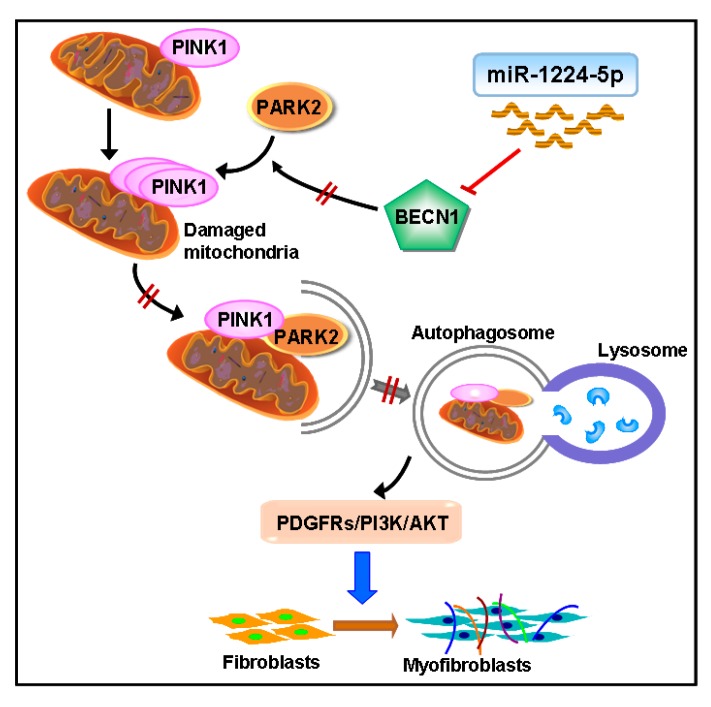
Schematic diagram showing the mechanisms by which miR-1224-5p regulates silica-induced pulmonary fibrosis. miR-1224-5p targets BECN1, which lead to block PARK2 translocation to mitochondria and impairing mitophagy activity, then accumulating damaged mitochondria. Moreover, the PDGFRs signaling pathway activation mediated by insufficient mitophagy could promote myofibroblast differentiation.

In conclusion, we identified miR-1224-5p as a novel therapeutic target in silica-induced pulmonary fibrosis. miR-1224-5p promoted silica-induced pulmonary fibrosis primarily by targeting BECN1 expression, thereby blocking PARK2 translocation to mitochondria and inducing the accumulation of damaged mitochondria. Moreover, the PDGFRs signaling pathway activation mediated by mitochondrial damage and insufficient mitophagy is responsible for myofibroblast differentiation (Figure 8). Our results represented a significant advance in our understanding of the regulation of mitochondrial damage and mitophagy activity by miRNAs in silica-induced pulmonary fibrosis and indicated that miR-1224-5p may represent a potential therapeutic target for silicosis.

## 4. Materials and Methods

### 4.1. Animals and Grouping

Wild-type C57BL/6 male mice (4–6 weeks old) were purchased from the Shanghai Laboratory Animal Center (SLAC, Shanghai, China). C57BL/6 mice were divided into 4 groups randomly (*n* = 6 in each group): saline, day 7, 14, and 28 silica groups. Mice in saline group were intratracheally administered 0.05 mL sterile saline as a negative control. Mice in day 7, 14, and 28 silica group were intratracheally administered 50 mg/kg silica (Sigma Aldrich, St. Louis, MO, USA) in 0.05 mL sterile saline. The lungs were harvested on day 7, 14 or 28 after silica instillation for further analysis.

For the miR-1224-5p downregulation mouse model of silica-induced pulmonary fibrosis, C57BL/6 male mice were divided into 4 groups randomly (*n* = 6 in each group): saline, silica, silica plus anta-NC and silica plus anta-1224-5p. The silica injection for pulmonary fibrosis was performed as described above. Either 5 nmol of miR-1224-5p antagomir (anta-1224-5p) or its negative control anta-NC (RiboBio Co., Ltd., Guangzhou, China) was co-administered with silica-suspended saline. Subsequently, 3 nmol of miR-1224-5p antagomir or anta-NC was injected via the tail vein weekly. Mice were subjected to pulmonary fibrosis analysis on day 28 after silica administration.

All animal procedures used in this study were approved by the Ethics Committee for Animal Experiments of Nanjing Medical University and were performed in accordance with the Guidelines for Animal Experimentation of Nanjing Medical University (Nanjing, China). Mice were housed under temperature- and humidity-controlled conditions (23 ± 2 °C), with a light/dark cycle of 12 h (07:00 to 19:00, 12 h) with food and water available ad libitum.

### 4.2. Fibroblast Culture and Treatment

The mouse fibroblasts (NIH/3T3) and the human fibroblasts (MRC-5) cell lines were obtained from American Type Culture Collection (ATCC, Manassas, VA, USA). Fibroblasts were maintained in Dulbecco’s Modified Eagle Medium (DMEM, Life Technologies/Gibco, Grand Island, NE, USA) with 10% fetal bovine serum (FBS, Life Technologies/Gibco, Grand Island, NE, USA), 100 U/mL penicillin and 100 µg/mL streptomycin (Beyotime Bio, Shanghai, China). The fibroblasts were plated (1 × 10^5^ cells) into 6-well plates overnight. The cells were then treated with 2 ng/mL TGF-β1 (eBioscience, affymetrix, Santa Clara, CA, USA) for 48 h, and the total RNA or protein was extracted according to the instructions.

### 4.3. Histological Analysis

Lung tissues from each treated mouse were subjected to stain with hematoxylin and eosin (H&E) staining. Briefly, lung tissues were fixed in 4% paraformaldehyde, embedded in paraffin overnight and then sectioned into 6 μm slices and stained using hematoxylin and eosin according to the manufacturers’ instructions. The panoramic scan system (3D HISTECH) was used for imaging histologic sections. To assess the lesions of lung tissues, a grading system of severity and distribution was utilized for each mouse according to the method previously described [42].

### 4.4. Quantitative Real Time PCR (qRT-PCR)

Total RNA from cells and mouse tissues was isolated using Trizol (Life Technologies/Ambion, Carlsbad, CA, USA). For miR-1224-5p measurement, 500 ng of total RNA was reverse-transcribed with specific primers. Reverse transcription was performed using the HiScript^®^ II Q Select RT SuperMix for qPCR Kit (Vazyme Biotech Co., Ltd., Nanjing, China). The AceQ^®^ qPCR SYBR^®^ Green Master Mix kit (Vazyme Biotech Co., Ltd., Nanjing, China) was used for miR-1224-5p amplification. The bulge-loop™ miRNA qRT-PCR Primer Sets (one RT primer and a pair of qPCR primers for each set) specific for miR-1224-5p were designed by RiboBio Co., Ltd. (Guangzhou, China). qRT-PCR analysis was performed using an ABI 7900HT Real-Time PCR System (Applied Biosystems). The relative expression levels of miR-1224-5p were normalized to the levels of U6 and calculated by the 2^−ΔΔ*C*t^ method.

### 4.5. Western Blot

The cells and lung tissues were lysed in lysis buffer (M-PER reagent for cells and T-PER reagent for tissues, Thermo Scientific, Waltham, MA, USA). Protein samples were electrophoresed on 10% polyacrylamide gradient gels, then transferred to nitrocellulose membranes. After blocked in 5% milk, the nitrocellulose membranes were incubated with primary antibodies at 4 °C overnight, then incubated with secondary antibody (Beyotime Bio, Shanghai, China) for 1 h. A ChemiDoc XRS+ imaging system (Bio-Rad Laboratories, Inc., Hercules, CA, USA) was used to perform membrane analysis. GAPDH was used as a loading control. Western blot bands were quantified using the Image J software (Scion, Torrance, CA, USA) (Supplemental experimental procedure).

Antibodies specific to PARK2 (No. ab179812), BECN1 (No. ab207612), Collagen I (No. ab34710), α-SMA (No. ab124964), SQSTM1/p62 (No. ab109012) and TOMM20 (No. ab186734) were purchased from Abcam. Antibodies specific to PINK1 (No. 6946), p-PDGFRα (No.2992), PDGFRα (No. 3174), p-PDGFRβ (No. 2227), PDGFRβ (No. 3169), p-PI3K p85 (No. 4228), PI3K p85 (No. 4257), p-AKT (No. 4060), AKT (No. 4691), Vimentin (No. 5741), and E-cadherin (No. 3195) were obtained from Cell Signaling Technology. Antibodies specific to GAPDH was obtained from Beyotime. (Supplemental experimental procedure)

### 4.6. Luciferase Assays

The wild type 3′UTR sequence of BECN1 that contains miR-1224-5p binding sites (BECN1 3′UTR-wt), and BECN1 mutant (BECN1 3′UTR-mut) were synthesized (GENEray Bio, Shanghai, China) and inserted into psiCHECK-2 plasmid. Luciferase assays were performed by seeding 5 × 10^4^ NIH/3T3 or MRC-5 cells per well in 24-well plates. Cells were co-transfected with BECN1 3′UTR wt or mut plasmid and miR-1224-5p mimic (miR-1224-5p) or mimic control (mimic-NC) using transfection reagent (RiboBio Co., Ltd., Guangzhou, China) according to the manufacturer’s instructions. The firefly and renilla luciferase activities in cells were measured via the dual-luciferase assay system after transfection for 24 h (Centro LB 960) and normalized as the quotient of renilla/firefly luciferase activities.

### 4.7. miRNA Inhibitor and siRNA Transfection

Approximately 1 × 10^5^ fibroblasts were seeded in 6-well plates for 24 h. Subsequently, 100 nM of miR-1224-5p inhibitor or inhibitor-NC (RiboBio Co., Ltd., Guangzhou, China) was transfected into cells for 24 h and then treated with 2 ng/mL TGF-β1. Cells were harvested for assays at 48 h after TGF-β1 treatment.

BECN1 siRNA (siBECN1) and PARK2 siRNA (siPARK2) were dissolved as suggested by the manufacturer (RiboBio Co., Ltd., Guangzhou, China). Non-targeting siRNA (si-NC) was used as the negative control. Fibroblasts were transfected with siBECN1 or siPARK2 and si-NC and then treated with TGF-β1 according to the methods described above.

### 4.8. Immunofluorescence Staining

Fibroblasts were cultured in a laser scanning confocal vessel. After miR-1224-5p inhibitor and siBECN1 co-transfection then TGF-β1 treatment, the cells were fixed with carbinol for 30 min and blocked with 5% BSA for 60 min. The cells were then incubated with primary antibody overnight at 4 °C and stained with secondary antibody in the dark. TOMM20 staining was used to perform confocal laser scanning microscopy analysis of mitochondria (ZEISS LSM 700). Immunofluorescence staining was performed for colocalization analysis of TOMM20 and PARK2 staining.

### 4.9. Co-Immunoprecipitation Assay

Fibroblasts were washed twice, harvested in PBS, and then lyzed in ice-cold IP lysis buffer containing 50 mM Tris, 1 mM EDTA, 0.5% NP-40, 150 mM NaCl, 10% glycerol and 1 mM PMSF for 30 min. Total cell extracts were centrifuged for 30 min at 12,000 rpm at 4 °C, then the protein A/G agarose beads (Beyotime Bio, Shanghai, China) or control IgG (Santa Cruz) was incubated with the supernatant for 1 h as a pretreatment. Lysates were incubated overnight with anti-PARK2 monoclonal antibody (Abcam, No. ab179812), anti-BECN1 monoclonal antibody (Abcam, No. ab207612) or control IgG and protein A/G agarose beads on rotary shaker at 4 °C. The immunoprecipitated complexes were collected by centrifugation at 3000 rpm for 5 min at 4 °C, and the beads were washed three times with IP lysis buffer and resuspended in 2 × SDS loading buffer. Subsequently, the immunoprecipitates complexes were eluted from the beads at 99 °C for 5 min. The eluted proteins were determined by Western blot analysis with indicated antibodies.

### 4.10. Transmission Electron Microscopy

Fibroblasts were transfected with miR-1224-5p inhibitor then treated with TGF-β1 for 48 h. After treatment, fibroblasts were fixed with 2.5% glutaraldehyde in phosphate buffer (pH 7.4) and postfixed for 2 h in 1% osmium tetroxide in phosphate buffer (pH 7.4). Cells were dehydrated with a graded series of acetone, then embedded and sectioned. Ultrathin sections were placed on copper mesh grids and stained with uranyl acetate and lead citrate and viewed on a Tecnai G2 Spirit Bio TWIN transmission electron microscope (FEI). The mouse lung tissues were fixed with 5% glutaraldehyde in phosphate buffer (pH 7.4), and the methods of treatment were the same as the above mentioned.

### 4.11. Mitochondria Isolation

Mitochondria were isolated from fibroblasts with a commercially available kit (Beyotime Bio, Shanghai, China) according to the manufacturer’s instructions.

### 4.12. Statistical Analysis

Data were presented as the means ± SD. Data were compared using independent-samples *t* tests (two groups) and one-way analysis of variance (ANOVA; more than two groups) with Dunnett’s test. A value of *p* < 0.05 was regarded as significant.

## Figures and Tables

**Table 1:** Effect of miR-1224-5p antagomir administration on lung histopathology.

Groups	Lesion Severity Grade						Average Severity Grade	Lesion Distribution Grade						Average Distribution Grade
	0	1	2	3	4	5		0	1	2	3	4	5	
Saline	6						0	6						0
Silica		1	1	1		3	3.5 ± 1.8 **		1	3	1		1	2.5 ± 1.4 **
Silica + anta-NC		1	2		1	2	3.2 ± 1.7 **		2	1	2		1	2.5 ± 1.5 **
Silica + anta-1224-5p	3	2			1		1.0 ± 1.5 ^##^	3	2		1			0.8 ± 1.2 ^##^

Values represent the mean ± SD of six for each group, ** *p* < 0.01 vs. the saline group, ^##^ *p* < 0.01 vs. the silica plus anta-NC group.

## Supplementary Materials

Supplementary materials can be found at www.mdpi.com/1422-0067/18/11/2357/s1.

## Abbreviations

TGF-β1	Transforming growth factor beta 1
BECN1	Beclin 1, autophagy-related
PARK2	Parkinson disease (autosomal recessive, juvenile) 2
PDGFR	Platelet-derived growth factor receptor
TGF-βRII	Transforming growth factor beta receptor 2
TNF-α	Tumor necrosis factor alpha
LPS	Lipopolysaccharide
α-SMA	Alpha-smooth muscle actin
PINK1	PTEN induced putative kinase 1
