# Supplementary material for: miR-1224-5p Mediates Mitochondrial Damage to Affect Silica-Induced Pulmonary Fibrosis by Targeting BECN1
Source: Int J Mol Sci. 2017 Nov 7;18(11):2357. doi: 10.3390/ijms18112357 (PMC5713326; doi:10.3390/ijms18112357)
Supplement: Supplementary file 1 [file ijms-18-02357-s001.pdf]

Supplementary Materials: miR-1224-5p Mediates Mitochondrial Damage to Affect Silica-Induced Pulmonary Fibrosis by Targeting BECN1

Qiuyun Wu, Tiantian Xu, Yi Liu, Yan Li, Jiali Yuan, Wenxi Yao, Qi Xu, Weiwen Yan and Chunhui Ni

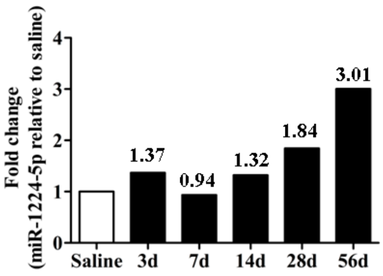

Figure S1. The fold changes of miR-1224-5p in C57BL/6 mouse lung tissues that harvested on days 3, 7, 14, 28 and 56 after the silica administration were determined by microRNA (miRNA, miR) microarray with total RNAs analysis.

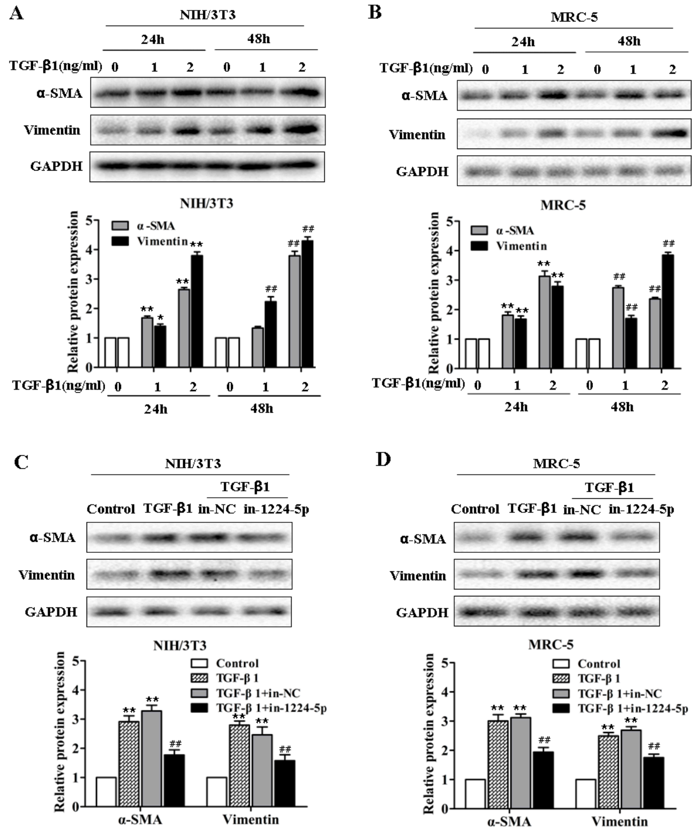

Figure S2. (**A**,**B**) Western blot and densitometric analysis of α-SMA and Vimentin expression in fibroblasts (NIH/3T3 and MRC-5) treated with different dose of TGF-β1 for 24 and 48 h, with * $p < 0.05$ and ** $p < 0.01$ vs. the 24 h control group, with ## $P < 0.01$ vs. the 48 h control group; (**C**,**D**) Western blot and densitometric analysis of α-SMA and Vimentin expression in fibroblasts (NIH/3T3 and MRC-5) transfected with miR-1224-5p inhibitor (in-1224-5p) or its negative control (in-NC) then treated with 2 ng/mL TGF-β1 for 48 h, with ** $p < 0.01$ vs. the control group and ## $p < 0.01$ vs. the TGF-β1 plus in-NC group. All data are expressed as the mean ± SD of at least three independent experiments.

Supplemental Experimental Procedure

Western Blot

The cells and lung tissues were lysed in cold lysis buffer (M-PER reagent for cells and T-PER reagent for tissues, Thermo Scientific) for 30 min. The supernatant were collected by centrifugation at 12,000 rpm for 15 min at 4 °C. The protein concentration was measured using a BCA protein assay kit (Beyotime Bio, Shanghai, China). A total of 80 μg protein samples were subjected to the SDS-PAGE on 10% polyacrylamide gradient gels at 60 V (constant voltage) for 3 h. The PVDF membranes (0.22 μm pore size, Millipore, Bedford, MA, USA) were incubated in methanol for about 1 min at room temperature, then the gels were transferred to PVDF membranes in transfer buffer at 200 mA (constant current) for 90 min. The membranes were blocked in 5% nonfat milk in Tris buffered saline containing 0.1% Tween 20 (TBST; 50 mM Tris-HCl (pH = 7.6), 150 mM NaCl, 0.1% Tween 20) at room temperature for at least 1 h. The primary antibodies were diluted at recommended proportion in 5% nonfat milk. The membranes were incubation with primary antibodies at 4 °C overnight, and then washed in TBST buffer for 15 min. After incubation with the appropriate secondary antibody (Beyotime Bio, Shanghai, China) diluted in 5% nonfat milk for 1 h at room temperature, the membrane were washed three times in TBST buffer (15 min for per time). The membrane analysis was performed via a ChemiDoc XRS+ imaging system (Bio-Rad Laboratories, Inc., Hercules, CA, USA). GAPDH was used as a loading control. Western blot bands were quantified using the Image J software.

Supplemental Experimental Data

Table S1. The densitometric analysis of Western blot in Figure 1C.

		**Saline**	**7d**	**14d**	**28d**
1	E-cadherin	8708.9	6796.1	3586.8	2685.3
	α-SMA	3067.4	5075.9	6097.1	8830.5
	Vimentin	4206.3	5004.2	5282.7	6209.6
	GAPDH	4853.5	4921.7	4201.1	4294.4
	Collagen I	2706.5	5760.8	9570.9	11,476.4
	GAPDH	11,388.8	15,969.7	12,874.9	13,175.2
2	E-cadherin	9031.8	8190.4	4367.7	1732.5
	α-SMA	2394.4	3890.0	6935.3	7884.1
	Vimentin	2597.6	3941.0	5160.8	5195.2
	GAPDH	9769.9	9731.3	10,753.7	8224.0
	Collagen I	3071.6	6209.1	10,563.8	14,627.2
	GAPDH	12,641.1	16,112.7	15,578.9	14,196.4
3	E-cadherin	4221.1	3186.4	3294.3	1871.1
	α-SMA	2868.9	4675.1	9735.5	9678.2
	Vimentin	3034.8	3017.1	5724.6	6051.7
	GAPDH	9135.1	7831.3	10,733.2	9241.8
	Collagen I	6944.7	8378.9	25,321.5	27,676.8
	GAPDH	12,745.9	12,148.9	13,553.8	14,043.4

Table S2. The densitometric analysis of Western blot in Figure 1E.

		**Saline**	**7d**	**14d**	**28d**
1	PINK1	4714.1	7845.1	2578.8	1743.0
	PARK2	9764.8	22,934.9	7196.7	3843.8
	p62	3293.7	4096.5	4531.0	6995.9
	GAPDH	5151.6	5308.4	4989.8	5237.6
2	PINK1	10,927.5	24,347.7	9042.8	8951.8
	PARK2	21,459.4	45,267.0	21,650.6	14,551.7
	p62	14,515.2	27,649.3	27,879.4	35,547.6
	GAPDH	26,242.0	28,973.6	25,998.4	27,115.6
3	PINK1	19,514.3	32,719.4	12,852.4	10,971.7
	PARK2	18,249.8	30,282.3	11,949.7	6209.6
	p62	24,016.4	26,403.6	29,885.5	42,439.5
	GAPDH	31,238.3	27,498.5	26,546.8	29,021.7

Table S3. The densitometric analysis of Western blot in Figure 2D.

		**Saline**	**Silica**	**Silica+anta-NC**	**Silica+anta-1224-5p**
1	E-cadherin	5775.4	2682.5	3003.5	5467.6
	α-SMA	3943.3	9109.2	9262.8	3794.7
	Vimentin	1958.2	4398.0	4763.7	2729.4
	GAPDH	6468.8	6587.0	6085.5	5793.8
	Collagen I	4213.0	14,672.3	17,715.5	5547.8
	GAPDH	7894.6	8829.6	9040.0	8984.8
2	E-cadherin	24,472.2	6864.4	6338.3	22,024.6
	α-SMA	3806.9	6359.7	7536.8	5195.1
	Vimentin	4556.9	8714.0	10,244.8	5390.6
	GAPDH	13,940.2	11,704.6	13,864.8	12,053.8
	Collagen I	7367.3	22,760.8	21,033.4	7566.5
	GAPDH	11,770.9	10,019.9	10,591.7	9197.8
3	E-cadherin	18,328.1	11,077.8	10,026.2	23,577.7
	α-SMA	3968.7	8652.8	10,006.3	6656.1
	Vimentin	4552.6	8346.8	9378.2	4517.7
	GAPDH	5186.1	4986.0	5325.1	5236.8
	Collagen I	3485.1	11,359.6	11,032.1	6109.9
	GAPDH	11,705.2	12,198.9	12,779.7	13,690.0

Table S4. The densitometric analysis of Western blot in Figure 2E.

		**Saline**	**Silica**	**Silica+anta-NC**	**Silica+anta-1224-5p**
1	PINK1	7963.1	3466.9	2800.6	9661.1
	PARK2	6887.9	3093.9	4506.9	8087.7
	p62	2326.6	5021.4	5849.0	2241.5
	GAPDH	6018.2	5372.9	5834.0	5598.3
2	PINK1	32181.3	16,664.1	22,189.8	36,381.8
	PARK2	5681.8	2766.9	1719.9	6016.7
	p62	2742.3	6096.8	6380.6	4089.7
	GAPDH	21,147.5	21,017.8	21,378.7	22,447.6
3	PINK1	17,678.0	5188.8	7453.4	24,227.0
	PARK2	22,287.5	6532.0	7093.4	18,284.3
	p62	4656.3	10,323.3	9082.9	6727.3
	GAPDH	18,864.4	21,359.7	17,679.0	19,070.9

Table S5. The densitometric analysis of Western blot in Figure 3D.

		Control	TGF-β1	TGF-β1+in-NC	TGF-β1+in-1224-5p
	NIH/3T3				
1	PINK1	18,160.0	5380.5	5474.5	19,462.8
	PARK2	18,586.1	7721.9	8110.1	15,028.9
	p62	12,382.3	22,124.5	17,202.0	7242.2
	GAPDH	15,490.6	13,169.2	12,805.0	12,641.1
2	PINK1	32,276.8	13,388.5	12,531.1	35,019.4
	PARK2	28,512.2	9878.3	12,267.6	26,665.9
	p62	2119.1	6149.7	4782.2	2471.4
	GAPDH	5345.4	6316.2	6695.1	5879.6
3	PINK1	29,411.9	15,742.8	13,915.9	32,097.6
	PARK2	13,542.3	6543.9	7721.8	13,543.7
	p62	4220.9	9672.4	9043.7	4819.4
	GAPDH	5035.6	5217.3	5109.3	5173.8
	MRC-5				
1	PINK1	23,132.3	8110.2	10,646.8	24,863.4
	PARK2	23,139.6	7302.1	9097.4	29,314.6
	p62	7053.2	14,046.5	17,112.1	8290.4
	GAPDH	10,394.3	10,885.7	11,422.4	12,865.2
2	PINK1	24,933.1	13,567.0	11,250.1	30,221.8
	PARK2	24,354.9	11,284.6	13,843.9	21,834.4
	p62	7836.3	13,243.8	17,225.8	9150.1
	GAPDH	6125.5	6358.7	6130.0	6122.4
3	PINK1	29,949.2	10,940.8	15,712.5	28,074.3
	PARK2	29,110.1	13,432.5	14,323.7	31,345.4
	p62	2094.1	4637.4	5292.5	2097.5
	GAPDH	27,942.3	28,699.0	27,825.1	26,070.4

Table S6. The densitometric analysis of Western blot in Figure 4C.

		Saline	7d	14d	28d
1	BECN1	21,783.8	20,597.6	11,838.5	6491.1
	GAPDH	23,264.0	23,373.5	21,075.0	23,199.7
2	BECN1	18,140.4	17,477.5	10,144.7	6674.4
	GAPDH	9066.6	8941.8	8160.3	8253.1
3	BECN1	13,733.0	16,412.1	6207.3	3296.8
	GAPDH	7629.6	7851.7	8781.1	9864.7

Table S7. The densitometric analysis of Western blot in Figure 4D.

		Saline	Silica	Silica+anta-NC	Silica+anta-1224-5p
1	BECN1	15,118.1	4579.1	6590.5	14,641.4
	GAPDH	17,226.9	17,261.9	18,664.8	16,946.8
2	BECN1	28,293.9	12,727.7	10,141.6	28,600.7
	GAPDH	11,745.6	12,612.5	14,371.3	13,247.0
3	BECN1	8634.2	2554.0	2782.9	6605.4
	GAPDH	12,204.8	12,785.4	11,211.6	9113.6

Table S8. The densitometric analysis of Western blot in Figure 4E.

		Control	TGF-β1	TGF-β1+in-NC	TGF-β1+in-1224-5p
	NIH/3T3				
1	BECN1	24,010.3	10,169.2	6469.1	23,651.5
	GAPDH	10,495.2	12,573.3	10,703.5	11,003.8
2	BECN1	25,321.5	11,628.2	9753.1	31,767.5
	GAPDH	27,270.2	26,049.7	30,032.4	28,500.9
3	BECN1	17,583.7	6784.4	8143.2	18,643.1
	GAPDH	33,642.2	29,792.9	33,479.0	34,928.1
	MRC-5				
1	BECN1	29,208.8	14,300.1	13,210.5	33,407.3
	GAPDH	12,759.5	14,053.9	13,628.9	15,971.9
2	BECN1	19,754.5	9587.3	8742.2	16,322.8
	GAPDH	18,515.9	14,174.3	18,823.2	17,404.3
3	BECN1	28,754.9	15,326.4	15,356.6	33,743.5
	GAPDH	34,590.0	34,757.6	31,094.1	36,717.5

Table S9. The densitometric analysis of Western blot in Figure 5B and 5C.

		Control	TGF-β1	TGF-β1+in-1224-5p	TGF-β1+in-1224-5p+siBECN1
	NIH/3T3				
1	PARK2	24,898.4	12,778.7	29,041.6	9231.0
	TOMM20	20,053.8	17,884.3	19,273.7	18,954.6
2	PARK2	34,045.7	18,784.7	31,145.9	15,114.2
	TOMM20	5272.7	7197.8	5225.4	6084.7
3	PARK2	12,998.8	5982.8	11,980.9	7005.0
	TOMM20	20,503.8	20,971.6	17,035.1	18,039.4
	MRC-5				
1	PARK2	14,397.2	5910.8	14,584.0	8813.4
	TOMM20	20,308.7	19,803.2	18,764.1	21,492.0
2	PARK2	12,332.9	4476.6	13,191.3	4598.0
	TOMM20	23,389.4	21,734.3	25,597.2	22,057.2
3	PARK2	9345.9	7313.6	11,179.2	6004.1
	TOMM20	6836.2	8596.3	7422.5	7097.0

Table S10. The densitometric analysis of Western blot in Figure 6A and 6B.

		**Control**	**TGF-β1**	**TGF-β1+si-NC**	**TGF-β1+siPARK2**
	NIH/3T3				
1	PARK2	37,324.7	21,724.1	20,777.7	10,257.7
	P62	10,553.5	15,944.8	21,302.7	27,384.6
	GAPDH	20,478.7	21,499.7	20,610.3	19,081.0
2	PARK2	8195.2	5856.6	3928.4	2762.6
	P62	16,463.0	27,611.2	32,518.8	37,111.1
	GAPDH	3938.4	3643.5	4194.3	4185.4
3	PARK2	18,952.9	11,697.5	12,875.4	4479.4
	P62	6712.9	8245.6	10,984.3	15,769.1
	GAPDH	13,304.3	13,045.3	14,557.4	15,737.5
	MRC-5				
1	PARK2	25,772.5	15,823.4	13,847.7	6538.8
	P62	10,439.0	15,538.0	17,063.7	24,003.2
	GAPDH	24,958.1	25,367.0	22,838.5	25,840.4
2	PARK2	21,703.5	10,043.1	10,369.7	6703.3
	P62	4575.1	9183.1	10,148.4	10,697.3
	GAPDH	11,710.7	12,318.2	13,287.9	11,853.3
3	PARK2	20,272.1	14,763.7	9441.6	2927.4
	P62	4204.5	9309.2	8301.4	10,537.3
	GAPDH	17,673.6	19,507.1	20,539.6	16,003.8

Table S11. The densitometric analysis of Western blot in Figure 7A.

		**Control**	**TGF-β1**	**TGF-β1+si-NC**	**TGF-β1+siPARK2**
1	PDGFR-α	12,274.6	12,777.1	12,639.3	13,893.3
	p-PDGFR-α	1199.4	2863.8	3251.0	5014.7
	PDGFR-β	9809.6	11,200.4	8589.5	8888.9
	p-PDGFR-β	2046.0	6012.2	5742.8	6524.8
	PI3K p85	30,669.4	30,511.7	26,734.0	28,461.6
	p-PI3K p85	7407.6	13,040.0	10,678.8	17,682.6
	Akt	16,666.7	17,618.5	19,298.0	20,179.1
	p-Akt	3458.5	6290.3	6463.2	8275.4
	α-SMA	1978.6	4257.4	4423.3	5370.6
	GAPDH	13,728.8	16,691.6	15,378.1	14,793.4
2	PDGFR-α	7140.2	7586.2	8024.1	7903.4
	p-PDGFR-α	3646.4	6736.5	7983.9	12,798.0
	PDGFR-β	14,144.9	15,207.8	18,852.3	14,756.5
	p-PDGFR-β	2173.3	7273.1	8169.4	9096.4
	PI3K p85	13,324.5	11,089.4	13,543.6	14,573.8
	p-PI3K p85	13,742.0	20,527.6	28,387.5	38,409.6
	Akt	8964.4	8653.3	7647.5	9467.3
	p-Akt	3479.5	5466.4	6176.8	9005.4
	α-SMA	1583.8	3042.7	3145.8	4423.3
	GAPDH	23,654.5	25,654.3	25,687.4	26,587.4
3	PDGFR-α	10,596.2	10,587.3	9447.9	9019.3
	p-PDGFR-α	3195.0	7657.7	7357.1	9884.8
	PDGFR-β	14,531.6	17,338.4	16,541.0	14,222.1
	p-PDGFR-β	2931.1	8363.5	9276.5	10,656.2
	PI3K p85	9565.4	10,034.6	11,436.4	10,865.4
	p-PI3K p85	7589.1	17,368.2	16,643.9	27,559.1
	Akt	13,945.4	16,534.5	14,576.5	15,438.6
	p-Akt	5467.9	9754.4	8954.6	12,335.7
	α-SMA	2431.2	4096.9	4761.3	6703.2
	GAPDH	8945.5	7834.6	9655.5	8756.4

Table S12. The densitometric analysis of Western blot in Figure 7B.

		**Control**	**TGF-β1**	**TGF-β1+si-NC**	**TGF-β1+siPARK2**
1	PDGFR-α	13,892.3	12,031.1	12,917.3	14,173.8
	p-PDGFR-α	6312.9	9103.9	10,517.5	15,847.7
	PDGFR-β	15,801.0	14,317.5	12,509.9	11,364.5
	p-PDGFR-β	8897.9	11,906.8	14,872.4	20,181.3
	PI3K p85	18,527.3	14,891.1	16,007.2	15,009.7
	p-PI3K p85	3315.0	5854.2	6176.1	7578.1
	Akt	35,059.2	34,298.3	32,824.5	35,681.3
	p-Akt	6506.1	11,553.7	10,388.4	16,780.6
	α-SMA	6275.9	8940.8	12,063.7	17,361.9
	GAPDH	15,975.2	15,689.1	17,470.3	18,840.9
2	PDGFR-α	6433.1	6397.8	5794.6	6538.5
	p-PDGFR-α	1899.6	3807.2	3158.6	6064.5
	PDGFR-β	10,590.5	8473.4	7835.6	9633.5
	p-PDGFR-β	2988.2	5017.2	3584.0	8455.6
	PI3K p85	12,564.9	10,345.4	11,356.7	13,343.7
	p-PI3K p85	8745.7	12,456.9	12,433.5	21,654.7
	Akt	15,432.5	14,564.3	16,755.7	18,569.4
	p-Akt	5753.5	8954.6	9356.2	15,436.2
	α-SMA	3889.8	6191.9	7070.0	9247.6
	GAPDH	13,324.5	11,235.6	15,345.3	16,476.4
3	PDGFR-α	4922.5	4561.6	4654.5	4327.2
	p-PDGFR-α	2359.5	4064.8	3365.1	5628.8
	PDGFR-β	17,060.7	19,033.3	19,259.6	18,135.4
	p-PDGFR-β	8959.3	19,365.4	20,090.9	23,421.6
	PI3K p85	12,435.6	16,554.6	14,658.1	17,543.4
	p-PI3K p85	3456.5	7753.7	7537.3	11,054.7
	Akt	7843.6	8934.5	7436.3	8543.6
	p-Akt	2456.5	6533.4	5468.6	7865.4
	α-SMA	3606.1	8176.0	7811.4	12,383.1
	GAPDH	12,543.5	15,765.4	14,650.3	16,765.3

Table S13. The densitometric analysis of Western blot in Figure 7C.

		Control	TGF-β1	TGF-β1+siPARK2+DMSO	TGF-β1+siPARK2+AG1296
1	PDGFR-α	22,202.0	21,203.8	19,951.3	18,145.7
	p-PDGFR-α	2812.4	5676.0	3914.6	1881.0
	PDGFR-β	21,041.9	16,457.7	17,659.7	18,456.8
	p-PDGFR-β	6437.4	11,589.3	14,008.1	11,534.2
	PI3K p85	20,632.5	22,891.6	24,257.7	23,801.1
	p-PI3K p85	2044.0	4132.4	4139.3	1901.1
	Akt	32,117.4	33,984.8	33,641.6	30,023.9
	p-Akt	3497.0	6991.3	6010.7	4660.2
	α-SMA	1961.2	3785.2	3233.7	3196.4
	GAPDH	25,161.0	24,002.0	22,282.7	29,236.6
2	PDGFR-α	5933.6	6649.6	6657.8	7615.8
	p-PDGFR-α	3547.8	7656.0	7980.4	5195.0
	PDGFR-β	24,976.5	28,465.4	29,454.3	26,547.9
	p-PDGFR-β	5394.3	12,994.4	12,665.3	9217.5
	PI3K p85	10,546.7	12,465.4	13,676.4	12,436.7
	p-PI3K p85	7264.1	13,113.4	16,995.8	10,499.1
	Akt	8389.5	9076.4	8954.3	8734.6
	p-Akt	14,490.9	23,312.1	26,098.0	15,685.2
	α-SMA	3035.4	7047.2	7274.6	4744.7
	GAPDH	15,670.4	17,443.8	16,576.6	17,576.4
3	PDGFR-α	9271.1	12,596.7	10,244.8	10,559.6
	p-PDGFR-α	5547.2	11,151.4	9575.3	5023.6
	PDGFR-β	15,578.4	15,540.6	13,259.6	14,674.9
	p-PDGFR-β	3893.9	6821.6	8123.5	5242.6
	PI3K p85	13,546.6	17,965.6	16,537.4	14,576.5
	p-PI3K p85	7304.7	17,130.5	18,319.2	10,925.8
	Akt	8954.6	8657.1	7057.3	9542.6
	p-Akt	11,779.8	22,549.2	21,064.3	16,831.9
	α-SMA	4861.0	7695.3	9077.8	6568.8
	GAPDH	18,976.8	19,845.3	20,546.7	21,456.4

Table S14. The densitometric analysis of Western blot in Figure 7D.

		Control	TGF-β1	TGF-β1+siPARK2+DMSO	TGF-β1+siPARK2+AG1296
1	PDGFR-α	13,103.0	15,924.3	16,587.1	11,356.7
	p-PDGFR-α	5474.5	14,545.5	16,626.9	5889.0
	PDGFR-β	18,555.0	17,593.2	18,445.9	15,124.8
	p-PDGFR-β	10,894.7	17,112.4	18,076.9	7514.1
	PI3K p85	21,736.3	20,948.3	18,244.9	20,096.9
	p-PI3K p85	2846.3	6380.2	6078.0	3822.8
	Akt	18,103.8	17,196.5	16,241.1	20,767.4
	p-Akt	5314.2	8840.2	8987.1	7819.7
	α-SMA	3732.1	8083.8	6306.6	3896.4
	GAPDH	7689.5	8876.5	8953.3	7843.7
2	PDGFR-α	11,012.8	12,787.4	12,575.6	12,413.5
	p-PDGFR-α	2538.3	6299.0	5430.5	4835.3
	PDGFR-β	9658.4	8956.3	8837.5	9845.7
	p-PDGFR-β	9434	19,303.4	18,490.0	11,591.4
	PI3K p85	15,236.6	17,854.7	15,654.7	16,653.6
	p-PI3K p85	8170.6	18,818.8	17,277.4	8926.9
	Akt	11,895.0	13,254.3	12,678.5	13,254.9
	p-Akt	16,230.1	36,347.1	30,515.6	19,340.4
	α-SMA	4758.6	7355.9	10,489.7	4931.4
	GAPDH	8945.4	7854.9	9854.3	8754.6
3	PDGFR-α	15,295.8	13,796.6	11,882.5	13,976.0
	p-PDGFR-α	7265.2	12,054.0	13,148.3	8333.9
	PDGFR-β	11,034.4	10,245.4	12,675.4	12,498.6
	p-PDGFR-β	13,982.6	23,249.7	27,146.9	15,607.6
	PI3K p85	6754.3	5424.9	5489.6	6054.3
	p-PI3K p85	8424.0	13,623.0	13,885.9	7976.8
	Akt	11,249.5	13,456.5	10,546.7	12,465.6
	p-Akt	16,323.6	31,256.0	25,381.5	15,232.2
	α-SMA	3011.0	7332.8	6411.8	4039.8
	GAPDH	7854.3	8965.3	9654.5	8324.5
